# Jarcho-Levin Syndrome With Fatal Respiratory Failure

**DOI:** 10.31486/toj.24.0111

**Published:** 2025

**Authors:** Sofien Atitallah, Wiem Ben Othmen, Rania Ben Rabeh, Nada Missaoui, Olfa Bouyahia, Sonia Mazigh, Salem Yahyaoui, Samir Boukthir

**Affiliations:** ^1^ Faculty of Medicine of Tunis, University of Tunis El Manar, Tunis, Tunisia; ^2^ Pediatric Department C, Bechir-Hamza Children's Hospital, Tunis, Tunisia

**Keywords:** *Congenital abnormalities*, *Jarcho-Levin syndrome*, *respiratory insufficiency*

## Abstract

**Background:**

First described in 1938, Jarcho-Levin syndrome is a rare genetic disorder characterized by multiple rib and vertebral anomalies that cause thoracic constriction and severe respiratory complications. Jarcho-Levin syndrome is associated with a high mortality rate.

**Case Report:**

We report the case of a 3-month-old male who was born with Jarcho-Levin syndrome to first-degree consanguineous parents. The infant presented with severe respiratory distress, scoliosis, thoracic cage deformity, and spinal dysraphism. Radiologic findings revealed multilevel vertebral segmentation defects and asymmetric rib deformities. Despite respiratory support, the infant's condition deteriorated, and he died from respiratory failure complicated by pneumonia at 7 months of age.

**Conclusion:**

This case highlights the life-threatening nature of Jarcho-Levin syndrome and emphasizes the critical role of early diagnosis in optimizing respiratory support and family planning. Genetic counseling is crucial and ideally recommended preconception or during early pregnancy for consanguineous families, although accessibility to counseling services varies widely. Despite advances in pediatric care, the prognosis for patients with Jarcho-Levin syndrome remains guarded, emphasizing the need for continued research into effective treatments and management strategies.

## INTRODUCTION

Costovertebral malformations, although rare, can be severely disabling when the rib and spinal abnormalities compromise respiratory function. Among the less common yet more severe forms of conditions that cause vertebral anomalies is Jarcho-Levin syndrome, a group of short-trunk skeletal dysplasias characterized by multiple segmentation defects of the vertebrae combined with rib anomalies.^1^

Saul Jarcho and Paul Levin first described the syndrome in 1938 in their report of 2 infants who exhibited multiple rib and vertebral anomalies that affected the entire spine, resulting in short-trunk dwarfism and a fatal outcome.^2^ Jarcho-Levin syndrome anomalies can cause respiratory insufficiency.^3^

We report the case of an infant diagnosed with Jarcho-Levin syndrome that was complicated with respiratory failure caused by the rib and spinal deformities.

## CASE REPORT

A 3-month-old male born to first-degree consanguineous parents was admitted with severe respiratory distress. The pregnancy had been irregularly monitored. A fetal ultrasound at 29 weeks of gestation revealed spinal anomalies, but the parents did not follow up for the remainder of the pregnancy. There was no family history of congenital anomalies or exposure to known teratogens during pregnancy. The infant was delivered at 37 weeks of gestation with a birth weight of 2,550 g.

The infant was hospitalized at birth for transient tachypnea of the newborn that resolved. The infant was hospitalized for 2 weeks, and oxygen therapy was administered. Jarcho-Levin syndrome was not diagnosed at that time. Upon discharge, the patient was referred to genetics for further consultation, but the parents did not follow up.

**Figure 1.**
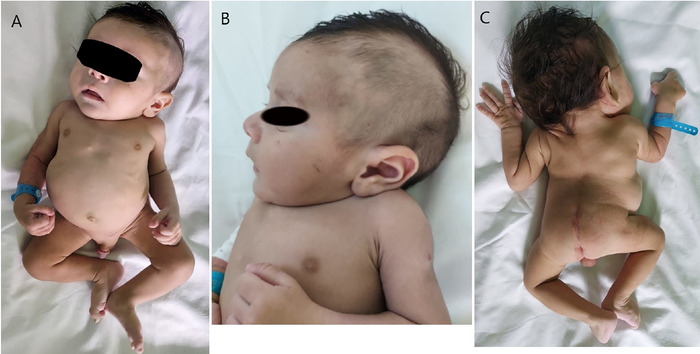
Physical examination images show (A) short-trunk dwarfism, malformed chest, and shortened lower limbs; (B) shortened neck; and (C) dysraphism with a neural tube closure defect, abnormal spinal curvature, asymmetry in the thoracic cage, and scoliosis. The rib cage has the distinctive crab-like appearance, with a protuberant abdomen.

Upon examination at presentation, the 3-month-old infant exhibited tachypnea with pronounced signs of respiratory distress. Physical examination revealed scoliosis, a low posterior hairline, thoracic cage deformity with a short trunk, short lower limbs, equinovarus deformity of the right foot, and spinal dysraphism with a neural tube closure defect extending from L1 to L5 (Figure 1).

**Figure 2.**
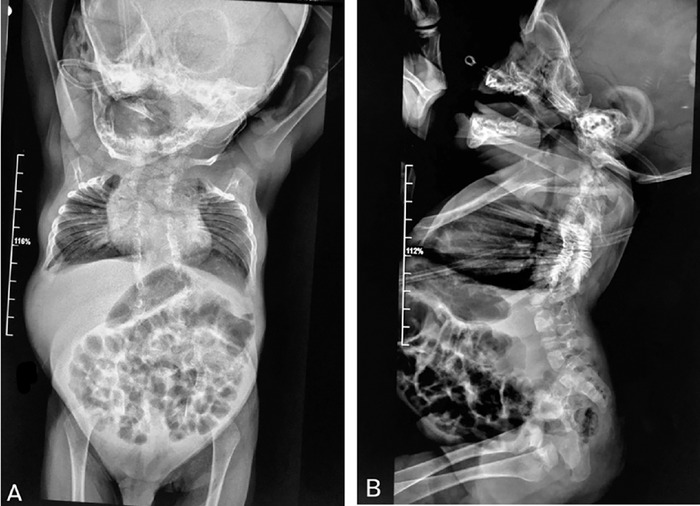
(A) Anteroposterior and (B) lateral radiographs of the thoracolumbar spine with kyphoscoliosis show abnormalities in the formation, segmentation, and curvature of the spine, predominantly at the thoracic level; abnormal closure of the posterior arches; and synostosis of the arches of the first and second ribs.

The infant was placed on a high-flow nasal cannula for respiratory support. Spinal ultrasound revealed a closed lipomyelomeningocele type of dysraphism with a low-lying spinal cord. Abdominal ultrasound showed a solitary left kidney, and cardiac ultrasound revealed a congenital anomaly of the left subclavian artery. X-rays showed multiple bilateral and asymmetric rib deformities, irregular rib fusions, and multilevel segmentation defects of the thoracic vertebrae (Figure 2).

Based on these clinical and radiologic findings, Jarcho-Levin syndrome was diagnosed. However, genetic testing could not be performed because of financial constraints.

The patient was hospitalized for 6 weeks. He received frequent respiratory assessments, and oxygen therapy was provided through high-flow nasal cannula support. Prognosis was discussed with the parents, and the patient was placed on continuous home oxygen therapy to maintain adequate oxygen saturation levels. Despite home oxygen therapy, during the next 4 months, the infant experienced 4 episodes of respiratory failure that were associated with pneumonia, and he required hospitalization each time for respiratory support. Each hospital stay was 2 to 6 weeks and included targeted antibiotic and high-flow nasal cannula therapy treatment for pneumonia.

During the infant's final hospitalization, noninvasive ventilation was used to provide advanced respiratory support. Broad-spectrum intravenous antibiotics were administered to treat pneumonia. The patient also received chest physiotherapy and nutritional support via a feeding tube. Despite these efforts, the patient's respiratory condition worsened, and he died from respiratory failure at 7 months of age.

## DISCUSSION

Both spondylothoracic dysostosis (STD) and spondylocostal dysostosis (SCD) involve abnormalities of the spine and ribs, and for more than half a century, there has been confusion regarding the distinction between the 2 conditions.^4^ Patients with SCD typically present with rib abnormalities such as fusion or the absence of ribs and with spine abnormalities such as block vertebrae or hemivertebrae. The clinical course in SCD can vary, but many individuals survive into adulthood. However, these abnormalities are more pronounced in patients with STD, with features such as cervicothoracic fusion and posterior spinal fusion. STD also leads to severe thoracic insufficiency and is associated with high infant mortality.^4,5^ However, a 2004 study estimated that more than 25% of patients with STD survive into adulthood despite thoracic insufficiency.^5^

Jarcho-Levin syndrome, now classified as a form of SCD,^4^ is characterized by multiple vertebral abnormalities at various levels of the spine, including butterfly vertebrae, hemivertebrae, and fused hypoplastic vertebrae, and the syndrome is often associated with rib abnormalities as well.^1^ Patients with Jarcho-Levin syndrome commonly present with respiratory distress because of thoracic constriction.^1^ The diagnosis is often suspected in patients with thoracic deformities and respiratory difficulties and can be confirmed with plain radiography.^1^

Jarcho-Levin syndrome has been linked to several genes, including DLL3 that is associated with the Notch pathway. In contrast, STD, or Lavy-Moseley syndrome, is thought to be associated with MESP2, which is also a Notch pathway gene.^1,6^

In our case, the patient exhibited multilevel vertebral segmentation defects causing scoliosis, accompanied by asymmetric rib deformity and thoracic constriction consistent with the typical findings of Jarcho-Levin syndrome. The lack of financial resources precluded determination of the specific gene. However, the clinical and radiologic features suggested an autosomal recessive pattern, as the parents did not exhibit any obvious malformations.

Jarcho-Levin syndrome is often associated with additional abnormalities, including hydrocephalus, spinal dysraphism, renal and urinary tract anomalies, clubfoot, and congenital heart defects.^7-9^ In our patient, the vertebral and rib abnormalities were associated with lipomyelomeningocele, solitary left kidney, and left subclavian artery anomaly.

The thoracic constriction caused by vertebral and rib abnormalities and the subsequent lung hypoplasia can lead to respiratory failure, respiratory compromise, and early death in children with Jarcho-Levin syndrome. As the child grows, the lungs cannot expand at the same rate, leading to increasing respiratory insufficiency. Respiratory compromise and infections are important concerns in affected individuals.^4^ Therefore, aggressive respiratory care and prompt treatment of pneumonia are essential in managing this condition.^4,7^

Surgical interventions with vertical expandable prosthetic titanium ribs have improved thoracic symmetry, controlled spinal deformity, and improved clinical respiratory function.^10,11^ Spinal fusion can be proposed to stabilize a progressive scoliotic deformity with pelvic obliquity.^7^

Prenatal diagnosis of Jarcho-Levin syndrome is possible as early as the twelfth week of gestation. Fetal ultrasound can aid in diagnosing Jarcho-Levin syndrome by detecting characteristic spine and chest defects, such as an irregular, short, pebble-like spine with malformed vertebrae, while amniotic fluid levels, limb length, and biparietal diameter often remain normal.^12^ Jarcho-Levin syndrome should be diagnosed at an early stage so management of the pregnancy can be improved and genetic counseling can be provided.^13^

## CONCLUSION

This case highlights the life-threatening respiratory complications of Jarcho-Levin syndrome. Although patients with SCD generally have a better prognosis than patients with STD, the respiratory complications resulting from SCD can be fatal. Early diagnosis and a respiratory management plan are crucial for optimizing outcomes. Genetic counseling is a valuable resource for consanguineous families to understand the risks associated with Jarcho-Levin syndrome.

## Notes

*This article meets the Accreditation Council for Graduate Medical Education and the American Board of Medical Specialties Maintenance of Certification competencies for Patient Care and Medical Knowledge.*
